# First person – Jie Sun

**DOI:** 10.1242/bio.051144

**Published:** 2020-03-24

## Abstract

First Person is a series of interviews with the first authors of a selection of papers published in Biology Open, helping early-career researchers promote themselves alongside their papers. Jie Sun is first author on ‘The transcription factor Spalt and human homologue SALL4 induce cell invasion via the dMyc-JNK pathway in *Drosophila*’, published in BiO. Jie conducted the research described in this article while a PhD student in Dr Jie Shen's lab at the Department of Entomology and MOA Key Laboratory for Monitory and Green Control of Crop Pest, China Agricultural University, Beijing, China. He is now a postdoc in the lab of Dr Wu-min Deng at Louisiana Cancer Research Center, New Orleans, LA, USA, investigating malignant rhabdoid childhood tumors using the *Drosophila* model.


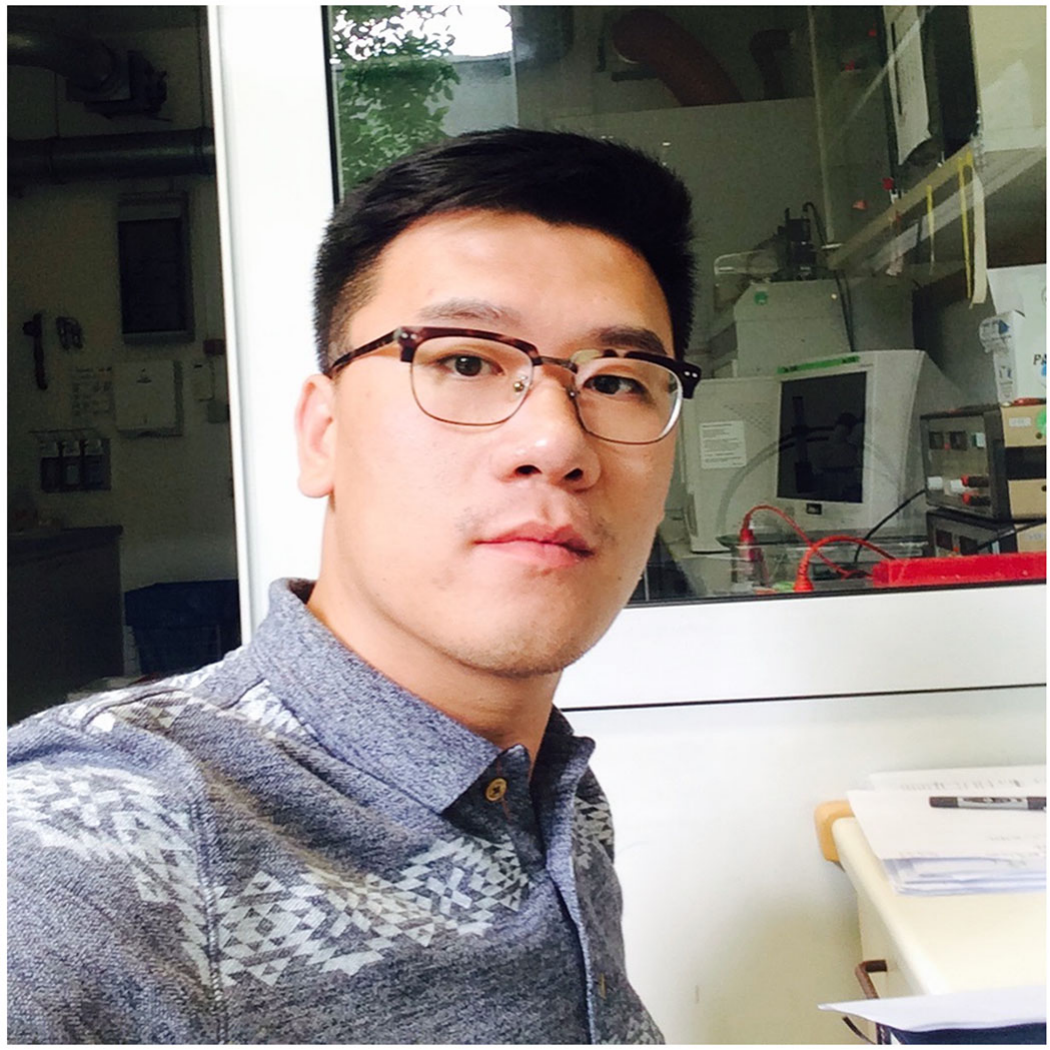


**Jie Sun**

**What is your scientific background and the general focus of your lab?**

In my research life in the past ten years, I have been devoted to using *Drosophila* as a model organism to study the mechanism of human diseases, whether in China, Germany or the United States. I build human psychiatric and cancer disease models in *Drosophila*. My PhD studies took place at China Agricultural University and Johannes Gutenberg University, focusing on the transcription factor-mediated signaling pathway and carcinogenesis of proto-oncogenes. Currently, at the medical school of Tulane University, I combine mammalian and *Drosophila* models to analyze tumor progression and the unique lipid metabolism that accompanies it. As a biological researcher, I am always intrigued by the uses of *Drosophila* as a model organism to study the molecular and cellular mechanisms underlying these human diseases.

**How would you explain the main findings of your paper to non-scientific family and friends?**

Due to the simplicity and stability of the *Drosophila* genome, it is often used to study gene function. In our paper, we used wing discs of *Drosophila* to analyze the malignant tumor invasion mechanism of epithelial cells. We focus on the functional study of *SALL4*, a key transcription factor in tumor metastasis. Both *Drosophila sal* and human *SALL4* transgenic flies generated migrating cells with invasive behavior in the *Drosophila* larval tissues. We revealed that *sal/SALL4*-induced cell invasion depends on dMyc-JNK signaling and is independent of the apoptotic pathway.

**What are the potential implications of these results for your field of research?**

Tumor metastasis, but not primary overgrowth, is the leading cause of mortality for cancer patients. Therefore, revealing the molecular mechanism of tumor cell invasion is of significance for tumor treatment. In human patients, the excessive activation of the transcription factor *SALL4* is sufficient to induce the occurrence and metastasis of malignant tumors. Here, we reveal the conserved role of *sal* and *SALL4* in invasive cell movement. We also linked the tumor metastasis mediated by *SALL4* with the JNK pathway, the key pathway of tumor invasion. These results provide new insights into the molecular mechanisms of *sal/SALL4*-induced cancer invasion and metastasis.

**What has surprised you the most while conducting your research?**

The *myc* gene is one of the most highly amplified oncogenes among many human cancers, and it promotes cancer progression and metastasis downstream of various signaling pathways including the Hippo, Notch and Wnt-APC pathways. These results are consistent with previous studies on *SALL4*, reporting that *SALL4* directly activates the oncogene *cMyc*. However, what's interesting is that we found that *dMyc* is repressed in *sal/SALL4-*expressing regions and introducing *dMyc* partially rescues cell invasion, indicating a repressive role of *dMyc* in tumor cell migration. These data suggest that *myc* has a bivalent role in regulating tumorigenesis and cell invasion.

“…we found that *dMyc* is repressed in *sal/SALL4-*expressing regions and introducing *dMyc* partially rescues cell invasion, indicating a repressive role of *dMyc* in tumor cell migration.”

**What, in your opinion, are some of the greatest achievements in your field and how has this influenced your research?**

Cancer is a complex disease that affects multiple organs. Genetic studies using *Drosophila* have explored the role of oncogenes and tumor suppressor genes, which promote tumor formation when imbalanced, making *Drosophila* a useful model for studying many aspects of transformation. *Drosophila* is not limited to mechanistic analysis; it also provides a fast and effective platform through which new drugs can be identified as candidate anticancer drugs. Furthermore, using *Drosophila* as a model means it is also possible to develop targeted therapies for individual patients by modeling the genetic complexity of cancer patients.

**What changes do you think could improve the professional lives of early-career scientists?**

I don't think being a scientist is an easy job compared to many professions. Scientific research is often full of difficulties, frustrations, and pressures, especially for early-career scientists. And science is the process of constantly discovering new knowledge and overthrowing misconceptions. Therefore, I think it is important for young scientists to be encouraged and supported as much as possible. This can give us the courage to boldly propose breakthrough ideas. Obviously having an excellent and patient mentor is definitely the ideal choice. In addition, I think that the current scientific community overemphasizes the importance of articles and impact factors, and only recognizes positive experimental results, which is detrimental to the development of young scholars. I think we should also recognize the significance of negative experimental results, which can provide reference for other scholars.

**Figure:**
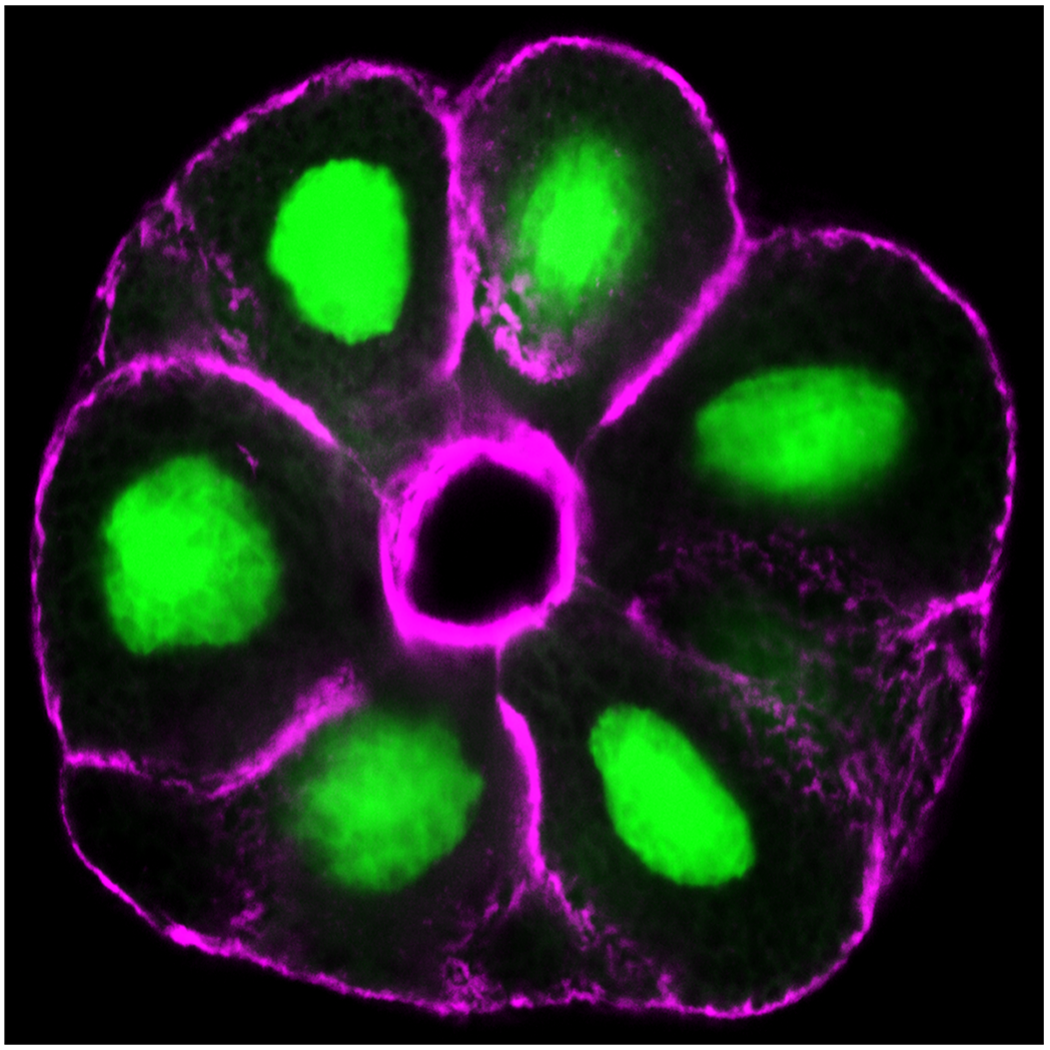
**Immunostaining of frozen sections of salivary glands of *Drosophila* larvae.**

“…it is important for young scientists to be encouraged and supported as much as possible. This can give us the courage to boldly propose breakthrough ideas.”

**What's next for you?**

I have already started my post-doctoral work, focusing on the use of *Drosophila* to address disease mechanisms and therapeutics, primarily for cancer. After that, I will further explore cancer treatment methods in combination with other traditional cancer tools, hoping to make my research of a more clinical value.
